# Comparative evaluation of the diagnostic value of HALP Score and systemic inflammatory immune index biomarkers in childhood appendicitis

**DOI:** 10.12669/pjms.41.9.12438

**Published:** 2025-09

**Authors:** Yeliz Kart, Emine Bilaloglu, Adnan Karaibrahimoglu

**Affiliations:** 1 Yeliz Kart Department of Pediatric Surgery, Faculty of Medicine, Suleyman Demirel University, Isparta, Turkey; 2 Emine Bilaloglu Department of Pediatric Surgery, Bayburt State Hospital, Bayburt, Turkey; 3 Adnan Karaibrahimoglu Department of Biostatistics and Medical Informatics, Faculty of Medicine, Suleyman Demirel University, Isparta, Turkey

**Keywords:** Appendicitis, Childhood, HALP score, Systemic Immune-Inflammation Index

## Abstract

**Objective:**

Abdominal pain is a frequent cause of pediatric emergency admissions. While appendicitis is the most common surgical cause, many non-surgical conditions also exist. Delays in diagnosis increase the risk of perforation. Biomarkers like the Systemic Immune Index (SII) and HALP Score have recently been used to predict prognosis in various conditions, especially cancer. This study aimed to evaluate the diagnostic value of SII and HALP scores in pediatric appendicitis, comparing them with each other and with other commonly used or proposed biomarkers.

**Methodology:**

Patients under the age of 18 who were hospitalized in the Pediatric Surgery Clinic of Süleyman Demirel University Faculty of Medicine Hospital with suspicion of appendicitis between 2011 and 2023 were included in this study. Demographic data, lab values, surgical notes and pathology results were reviewed. Patients were grouped as nonspecific abdominal pain (NAP), acute appendicitis (AA) and complicated appendicitis (CA).

**Results:**

Among 719 patients, 212 had NAP, 403 had AA and 104 had CA. SII was significantly higher in AA compared to NAP and rose with disease severity (p<0.001). HALP scores differed significantly among all groups (p<0.001). SII better distinguished AA from NAP, while HALP was more effective in identifying CA from AA. Increased SII and decreased HALP were linked to higher risk of AA and CA.

**Conclusions:**

No single marker reliably differentiates NAP, AA and CA. This study suggests that SII is useful in identifying AA, while HALP better detects CA, indicating both markers may aid in the diagnostic process of pediatric abdominal pain.

## INTRODUCTION

Emergency department visits due to abdominal pain are common in childhood and while the most common surgical cause of abdominal pain in this age group is acute appendicitis (AA), there are also many nonsurgical causes. The diagnosis of appendicitis in children can be difficult due to reasons such as incomplete anamnesis and lack of complete physical examination. In pediatric patients, there is a negative laparotomy probability ranging from 8% to 30%. In order to avoid unnecessary appendectomies, many diseases that can mimic AA, such as mesenteric adenitis, gastroenteritis, upper respiratory tract infections, pneumonia and urinary tract infections should be considered in the differential diagnosis.^1^ If the diagnosis is uncertain, the probability of appendiceal perforation increases as the time it takes for clinical findings to become apparent. The risk of perforation in AA varies from 23% to 73% in young children and reaches up to 100% in infants under one year old, while it is 16%-39% in adults.^2^

Physical examination, imaging methods and blood tests are used for diagnosing appendicitis. Simple, widely available and inexpensive inflammatory tests are still used to support the clinical diagnosis of AA as it causes a systemic inflammatory response. The most preferred markers are C-reactive protein (CRP) level, white blood cell (WBC) count and neutrophil percentage (NP). It has lately been reported that the measurements received from blood tests are beneficial in diagnosing AA. Of these, the most commonly used are the neutrophil-to-lymphocyte ratio (NLR), serum sodium level, mean platelet volume (MPV) and platelet-to-lymphocyte ratio (PLR).^2^

Recently, several biomarkers have been described in the literature and studies have particularly been published to define the prognostic features of cancer patients. Systemic immune-inflammation index (SII) is the first of these biomarkers being a new marker of inflammation and calculated as the combination of PLT, neutrophil and lymphocyte counts (PLT × neutrophil/lymphocyte counts). Hu et al. first defined SII in 2014 to evaluate the prognosis of patients with hepatocellular cancer.^3^ In recent years, various studies have been reported on the progression and prognosis of neurological and inflammatory diseases and cancers.^4^ Chen et al. have developed another marker, the HALP score, to predict the prognosis of gastric carcinoma. It is calculated as [hemoglobin (g/L) × albumin (g/L) × lymphocytes (/L)]/platelets (/L). The components of the HALP score are related to the immune and nutritional status of cancer. Studies investigating the HALP score, especially in inflammatory diseases requiring surgical intervention, are limited.^5^

We hypothesized that the difficulty in diagnosing acute appendicitis in pediatric patients compared to adults, particularly when a definitive diagnosis cannot be made with ultrasonography, suggests that the SII (Systemic Inflammatory Index) and HALP (Hemoglobin, Albumin, Lymphocyte, Platelet) scores could be potential diagnostic tools for children with appendicitis to avoid the existing radiation risk of computed tomography and unnecessary laparotomies. Therefore, this study aimed to investigate the diagnostic value of these biomarkers in children with appendicitis by comparing SII and HALP scores with each other and with other biomarkers routinely used or proposed to be useful in the diagnosis.

## METHODOLOGY

Patients under the age of 18 who were hospitalized in the Pediatric Surgery Clinic of Süleyman Demirel University Faculty of Medicine Hospital with suspicion of appendicitis between 2011 and 2023 were included in this study. The medical records of the patients were retrospectively analyzed for demographics, medical history, laboratory findings measured on admission, operation notes and pathological reports. Patients were classified as nonspecific abdominal pain (NSA), acute appendicitis (AA) and complicated appendicitis (CA).

The 719 patients undergoing appendectomy and whose diagnosis was confirmed by histopathological examination were included in the AA group. The NAP group (n=212) consisted of the patients hospitalized because of the suspected appendicitis, but completely recovered after the follow-up and included without appendectomy. The groups were compared in terms of age, sex, WBC count, PLR, NLR, CRP, SII, HALP score and sodium level. The patients having incomplete medical records, appendiceal neoplasm, hematological and inflammatory diseases, infection within the last three months, malabsorption, malignancy and a history of antibiotic usage were excluded from the study.

### Ethical Approval:

The study was conducted in accordance with the declaration of Helsinki and was approved by the local Ethical Committee (Approval no: 72867572-050.01.04; dated December 29, 2023).

### Statistical analysis:

The analyses of the study were performed by SPSS 27.0 (IBM InCorp, Armonk, NY, USA) software. The descriptive statistics were presented as frequency (percentage) for categorical variables and Mean ± SD for numerical variables. The Kolmogorov-Smirnov test was used to evaluate the normal distribution pattern of continuous data. The relationship between the categorical variables was determined by the Chi-square test and the difference between the patient groups for continuous variables was analyzed by the Kruskal-Wallis test with the K-W critical difference post-hoc test. ROC analysis was performed to determine the predictability of SII and HALP scores among the groups. The optimum cut-off value, sensitivity, specificity and predictive values of the studied biomarkers were identified by the receiver operation characteristic (ROC) analysis with 95 % CI. The area under the ROC curve (AUC) from 0.5 to 0.7 is considered as low, 0.7 to 0.8 acceptable, 0.8 to 0.9 excellent and more than 0.9 as outstanding diagnostic value. In order to determine the effect of demographical characteristics and indices on the groups, multinomial logistic regression models were established for patient group categories. A p<0.05 value was considered as statistically significant result for 5% Type-I error.

## RESULTS

A total of 719 patients were included in the study: 212 in the NAP group, 403 in the AA group and 104 in the CAP group. A male predominance was seen in all groups, with a male-to-female ratio of 1.1 in the NAP group, 1.8 in the AA group and 1.9 in the CA group. The mean age was 10.17 ± 3.25 in NAP, 10.97 ± 3.42 in AA and 9.82 ± 3.79 years in CA. The mean age was 10.17 ± 3.25 years in the NAP group, 10.97 ± 3.42 years in the AA group and 9.82 ± 3.79 years in the CA group and the mean age of the CA group was significantly lower compared with both the NAP and AA groups (p=0.001). However, there was no significant difference between the NAP and AA groups in terms of mean age (p>0.05).

A comparison of the laboratory parameters of the groups is shown in Table-I. The mean serum WBC value was significantly higher than those in the AA and CA groups when compared to the NAP group (p<0.001), however, no significant difference was observed between the CA and the AA groups (p=0.356). Serum CRP values were significantly different among all groups (p<0.001). Serum sodium levels decreased significantly in the CA group when compared to AA and NAP groups (p<0.001), however, no significant difference was found between NAP and AA (p=0.283). The NLR and PLR values varied significantly between NAP and AA groups (p<0.001) and AA and CA groups (p1=0.038, p2=0.001). The SII value was significantly higher in the AA group than in the NAP group (p<0.001) and increased with the severity of appendicitis (p=0.002). The HALP score was significantly different among all groups (p<0.001).

**Table-I:** Comparison of the laboratory parameters of the study population

	NAP (n=212)	AA (n=403)	CA (n=104)	p
WBC (μL)	11475±4564^a,b^	15799±4731^a^	16892±6029^b^	<0.001*
CRP (mg/L)	20.75±33.78^a,b^	32.33±42.47^a,c^	139.53±75.71^b,c^	<0.001*
Sodium mmol/L)	136.97±2.41^a^	136.68±2.47^b^	134.47±2.96^a,b^	<0.001*
NLR	6.33±6.48^a,b^	11.24±8.49^a,c^	13.18±8.72^b,c^	<0.001*
PLR	187.80±126.34^a,b^	232.11±138.82^a,c^	292.94±172.08^b,c^	<0.001*
SII	1797±1758^a,b^	3180±2478^a,c^	4163±2987 ^b,c^	<0.001*
HALP	0.44±0.23 ^a,b^	0.34±0.17 ^a,c^	0.21±0.11 ^b,c^	<0.001*

* : significant at 0.05 level according to Kruskal-Walli tests, ^a,b,c^: Same superscript letters denote the significant pairwise comparisons at 0.05 level according to K-W critical difference post-hoc test.

**Fig.1A:**
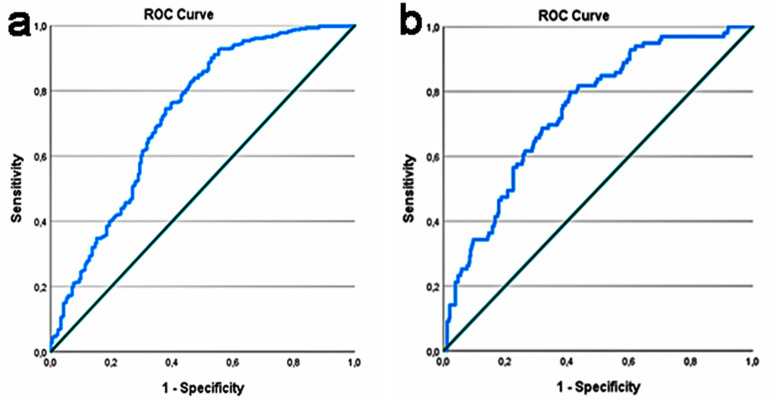
ROC curve of SII to distinguish AA from NAP, Fig.1B: ROC curve of HALP score to distinguish CA from AA.

The diagnostic performance of the SII value (AUC: 0.724; p<0.001) was more successful in distinguishing AA from NAP than the HALP score (AUC: 0.636; p<0.001). The optimum cut-off value for the SII was calculated as 928 (Sensitivity: 91.1% and Specificity: 46.2%), while it was 0.515 for the HALP score (Sensitivity: 87.7% and Specificity: 38.3%) (Fig.1A). It was observed that the HALP score (AUC: 0.740; p<0.001) was more successful in differentiating CA from AA than the SII (AUC: 0.619; p<0.001) (Fig.1B). The cut-off value for the HALP score was 0.269 (Sensitivity: 79.8% and Specificity: 59.1%), while it was 2628 for the SII (Sensitivity: 67.3% and Specificity: 53.1%).

A logistic regression model was created in order to calculate the risk values of SII and HALP score indices on appendicitis groups. It was also seen that SII and HALP score had a significant effect in the model created by taking the NAP group as the reference group (-2LL=1264.49; p<0.001). Increasing SII and decreasing HALP score was found to have a higher risk of acute and complicated appendicitis (Table-II).

**Table-II:** Effects of indices on appendicitis groups.

Groups	Parameters	Beta	p	OR (95% CI)
Acute Appendicitis	SII	0.012	<0.001*	1.012 (1.005-1.028)
	HALP	-3.070	<0.001*	21.730 (7.936-58.823)
Complicated Appendicitis	SII	0.009	<0.001*	1.009 (1.001-1.017)
	HALP	-3.300	<0.001*	27.112 (8.624-60.357)

* : significant at 0.05 level according to Multinomial Logistics Regression analysis

## DISCUSSION

Childhood appendicitis has always challenged clinicians since it can lead surgeons to misdiagnose and undergo negative exploration, while the delay in diagnosis may cause high morbidity and mortality because of the complication. Since appendicitis is an inflammatory disease, various biomarkers have been investigated for their usefulness in diagnosis. It was tried to reach the most definitive diagnosis with examination findings, radiological imaging methods and biomarkers obtained as a result of blood tests. Ultrasonography and computed tomography (CT) scanning tests can assist in the preoperative diagnosis of acute abdomen; however, these methods may not always be readily available in all primary healthcare facilities. Although imaging studies have been reported to aid in the diagnosis of appendicitis, the results of diagnostic imaging may not always provide a straightforward answer to completely rule out or confirm acute appendicitis in children, and the diagnostic accuracy of ultrasound can lead to false results due to the skill of the person performing the imaging. This situation may not provide sufficient confidence in diagnosing acute appendicitis based on the reports of imaging tests.^6^

Among the etiologies of abdominal pain, appendicitis is considered the most common cause of acute abdomen across all age groups.^7^ In this study, the mean age of patients with AA was 10.97 ± 3.42 years. The mean age of patients in the CA group was significantly lower (9.82 ± 3.79 years) than that in the AA group. It occurs in approximately 7%-8% of the population throughout life and is most frequently seen between the ages of 10 and 20.^2^,^8^ Our findings are consistent with the literature, in which the average age is also reported as 10-11 years. Appendicitis is less common in preschool-aged children and is often diagnosed at the perforation stage. Male patients constitute the majority of pediatric appendicitis cases^2^ and our study similarly found a higher incidence of appendicitis in males.

The serum WBC count was significantly elevated in both the AA and CA groups compared to the NAP group; however, there was no significant difference in WBC levels between the CA and AA groups. Although WBC is a frequently used biomarker in diagnosing AA, it lacks utility in differentiating between CA and AA, which aligns with our results.^2^,^9^ Increases in neutrophils or decreases in lymphocytes and platelets during appendix inflammation result in elevated NLR and PLR. These ratios were significantly higher in both the AA and CA groups compared to the NAP group, consistent with the literatüre.^2^,^6^,^9^

C-reactive protein (CRP), a key inflammatory marker, has been considered a useful indicator of appendicitis severity. In our study and in the literature, CRP levels were significantly higher in CA patients than in AA patients.^2^,^10^,^11^ Furthermore, serum sodium levels were significantly lower in the CA group compared to both the AA and NAP groups; however, no significant difference was observed between the AA and NAP groups.^2^,^6^,^9^

Recent studies have identified an association between the SII and various chronic and acute inflammatory conditions.^4^,^10^,^11^ High SII values are generally associated with poor outcomes in malignancies, cardiovascular diseases and autoimmune disorders.^10^ In our study, SII value was shown to be significant to distinguish AA from NAP. The diagnostic performance of SII was found to be comparable to that of NLR. Tekeli et al suggested that SII may help distinguish AA from CA, but our study showed lower accuracy rates for this distinction.^10^ In our results, while SII was effective in differentiating AA from NAP, its AUC was lower for distinguishing AA from CA.

The HALP score, which incorporates hemoglobin, albumin, lymphocyte and platelet levels, provides important information on the patient’s immunological and nutritional status. Previous studies have shown that a low HALP score is associated with poor prognosis in cancer patients.^12^-^18^ In our study, the HALP score was significantly lower in the CA group, likely due to more severe and prolonged inflammation. Notably, the HALP score was more effective than the SII in differentiating CA from AA, with higher sensitivity and specificity. Sarıdaş et al. reported that both SII and HALP scores were useful for distinguishing CA from AA in adult patients,^17^ while Benli and Tazeoğlu found a correlation between low HALP scores and complicated appendicitis in adults.^18^ Our findings support the usefulness of the HALP score in distinguishing CA from AA, in line with previous literature.

### Limitations:

Firstly, as it is a single-center study, the sample size is limited. Further multicenter studies involving a larger number of patients are needed. Secondly, since this study was conducted in a pediatric patient population, additional studies including adult patients would be beneficial.

## CONCLUSION

There is still no parameter with a high diagnostic value in making a distinction between NAP, AA and CA. In pediatric patients, making these distinctions is much more difficult. This study was carried out on pediatric patients and it was seen that SII was more effective in differentiating AA from NAP, but its effect in differentiating AA from CA was lower. We also determined that the HALP score was more effective in distinguishing the CA group from AA. Considering all these data, using these scores together can be guiding for the clinician, especially in cases where imaging methods are inadequate. These biomarkers have hardly been defined. There are limited studies in literature. Further studies are needed about their usefulness in the diagnosis of the appendicitis in pediatric patients.

### Authors’ Contribution:

**YK and EB:** Conceptualization. Preparation of manuscript.

**YK, EB and AK:** Data curation, analysis.

All authors have read and approved the final version of the article and are responsible for the accuracy and integrity of the work.
